# HES1 is required for mouse fetal hematopoiesis

**DOI:** 10.1186/s13287-024-03836-8

**Published:** 2024-07-29

**Authors:** Anthony Z. Zhu, Zhilin Ma, Emily V. Wolff, Zichen Lin, Zhenxia J. Gao, Xue Li, Wei Du

**Affiliations:** 1 Division of Hematology and Oncology, University of Pittsburgh School of Medicine, 5117 Center Ave, Pittsburgh, PA 15213 USA; 2 UPMC Hillman Cancer Center, Pittsburgh, PA 15213 USA; 3 Department of Biological Sciences, University of Notre Dame, Notre Dame, IN USA; 4 Master of Science in Medical Science, Boston University School of Medicine Graduate Master Program, Boston, MA USA; 5 Institute for Brain Research and Rehabilitation, South China Normal University, Guangzhou, China

**Keywords:** Fetal hematopoiesis, HES1, Fetal liver, Hematopoietic stem progenitor cells, Stem cell quiescence, Apoptosis

## Abstract

**Background:**

Hematopoiesis in mammal is a complex and highly regulated process in which hematopoietic stem cells (HSCs) give rise to all types of differentiated blood cells. Previous studies have shown that hairy and enhancer of split (HES) repressors are essential regulators of adult HSC development downstream of Notch signaling.

**Methods:**

In this study, we investigated the role of HES1, a member of HES family, in fetal hematopoiesis using an embryonic hematopoietic specific *Hes1* conditional knockout mouse model by using phenotypic flow cytometry, histopathology analysis, and functional in vitro colony forming unit (CFU) assay and in vivo bone marrow transplant (BMT) assay.

**Results:**

We found that loss of *Hes1* in early embryonic stage leads to smaller embryos and fetal livers, decreases hematopoietic stem progenitor cell (HSPC) pool, results in defective multi-lineage differentiation. Functionally, fetal hematopoietic cells deficient for *Hes1* exhibit reduced in vitro progenitor activity and compromised in vivo repopulation capacity in the transplanted recipients. Further analysis shows that fetal hematopoiesis defects in *Hes1*^*fl/fl*^*Flt3Cre* embryos are resulted from decreased proliferation and elevated apoptosis, associated with de-repressed HES1 targets, p27 and PTEN in *Hes1*-KO fetal HSPCs. Finally, pharmacological inhibition of p27 or PTEN improves fetal HSPCs function both in vitro and in vivo.

**Conclusion:**

Together, our findings reveal a previously unappreciated role for HES1 in regulating fetal hematopoiesis, and provide new insight into the differences between fetal and adult HSC maintenance.

**Supplementary Information:**

The online version contains supplementary material available at 10.1186/s13287-024-03836-8.

## Introduction

Hairy Enhancer of Split 1 (HES1) is a basic helix-loop-helix (HLH) transcriptional repressor [1] and an evolutionarily conserved target of Notch signaling [2, 3]. It plays an essential role in the development of many organs by promoting the maintenance of stem/progenitor cells, controlling the reversibility of cellular quiescence, and regulating both cell fate decisions and the timing of several developmental events [4, 5]. HES1 is more than a repressor. For example, HES1 amplifies RUNX2 (Runt-related transcription factor 2) expression by cooperating with retinoblastoma protein (pRb) through its WRPW domain [6]. In addition, HES1 uses basic helix-loop-helix (bHLH) domain and orange domain to bind with STAT3 and activates STAT3 phosphorylation [7]. Other unknown function of HES1 remains to be elucidated.

Blood cells are continuously produced from a pool of progenitors that derived from HSCs. Balanced stem cell self-renewal and initiation of lineage specification program is essential for the development and homeostasis of the hematopoietic system [8]. Embryonic hematopoiesis begins in the yolk sac and transitions to definitive hematopoiesis in the fetal liver [9]. On embryonic day E9.5 ∼ 10.5 in murine development, hematopoietic stem cells (HSCs) migrate from their origin sites to the fetal liver for expansion [10–12], before terminal migrating to the bone marrow (BM) on E16.5 to 17.5 [10–13]. This transient residence in the fetal liver is essential for the maturation of adult HSCs and functional adult hematopoiesis [12–14]. It has been shown that fetal hematopoietic progenitors are functionally distinct from adult progenitors [15]. For example, murine fetal progenitors exhibit greater engraftment efficiency, biased lineage differentiation, and altered susceptibility to transformation compared with adult counterparts [16–19]. Analogous studies in humans showed that fetal and cord blood CD34^+^ cells are more proliferative and have a greater propensity to form myeloid colonies in methylcellulose culture than adult cells [20, 21]. Although fetal liver of *Hes1*-deficient embryos had been previously analyzed [22], neither HSC phenotype nor function were investigated. A recent study showed that cumulative deletion of *Hes1* and *Hes5* alleles leads to the generation of nonfunctional hematopoietic progenitors in the developing aorta-gonad-mesonephros (AGM) and the total absence of HSC activity [23]. Our previous studies have demonstrated that HES1 is dispensable for steady state adult hematopoiesis [4]. However, the precise role of HES1 in regulating embryonic hematopoiesis remains elusive.

Both p27 (cyclin-dependent kinase inhibitor 1B; also known as p27^KIP1^) and PTEN (phosphatase and tension homolog deleted on chromosome 10) are known HES1 targets [24, 25]. p27^KIP1^ regulates the G1 to S-phase transition by binding to and regulating the activity of cyclin-dependent kinases [26]. Tumor suppressor, PTEN acts as a phosphatase for PIP3 and negatively regulates the PI3K/AKT pathway. However, the connection between HES1 and downstream factors, p27 and PTEN in fetal hematopoietic tissues/system are not known. Using a fetal hematopoietic *Hes1* specific knockout strain, here we show that loss of *Hes1* affects fetal hematopoiesis possibly through de-repressing the two HES1 downstream factors, therefore subsequently suppressing HSC proliferation and increasing HSC death. Together, our studies reveal a novel role for HES1 in regulating hematopoiesis during embryonic development, and add another layer of understanding the distinct HES1 functions in modulating gene expression at different stages of development.

## Materials and methods

### Mice and treatment

Heterozygous *Hes1*^*fl/+*^ mice [4, 27] were interbred with *Flt3Cre* mice [28, 29] to generate *Hes1*^*fl/fl*^*Flt3Cre* and *Hes1*^*fl/fl*^ littermates. This *Flt3Cre* strain allows reliable deletion of *Hes1* in multipotent progenitors (MPPs) at E10.5. 8 ∼ 12-week-old BoyJ mice were used as bone marrow transplant (BMT) recipients. Animals including BoyJ recipient mice were maintained in the animal barrier facility at Division of Laboratory Animal Resources (DLAR) at University of Pittsburgh. All animal experiments were carried out in accordance with the National Institutes of Health Guidelines for the Care and Use of Laboratory Animals and approved by the Institutional Animal Care and Use Committee (IACUC) of University of Pittsburgh.

Fetal liver cells isolated from *Hes1*^*fl/fl*^*Flt3Cre* or *Hes1*^*fl/fl*^ embryo were cultured in stem cell culture medium (IMEM supplemented with 1% glutamine, 20% FBS, 50 ng/ml m-SCF, 20 ng/ml mTPO, and 1% Pen-Strep; [30]) in the presence or absence of 2 μM of p27 inhibitor, SJ403 (full name SJ572403; MedChemExpress; [31, 32]); or 100 μmol/L of PTEN inhibitor (bpV(HOpic) from Sigma-Aldrich; [33, 34]), for 24 h followed by flow cytometry analysis, CFU or bone marrow transplant (BMT).

### Flow cytometry analysis

Single-cell suspensions were prepared from the fetal livers and cells were stained with saturating concentrations of Pacific Blue, FITC, PE, PE-Cy7, PerCP, APC, APC-Cy7 or e-Fluor780 conjugated antibodies in PBS containing 0.1% BSA and 0.05% NaN3 at 4 ºC for 30 min, then washed with PBS and subsequently analyzed using a BD Biosciences FACS Fortessa and FCS express software. For the analysis of hematopoietic stem and progenitor (HSPC) populations, 1 × 10^6^ fetal liver cells were stained with a biotinylated antibody cocktail against lineage markers CD3, CD8a, CD19, B220, Gr1, CD11b, CD11c, Ter119 (BD Bioscience, San Diego, CA; Cat: # 559971) followed by streptavidin PerCP-Cy5.5 (BioLegend, San Diego, CA; Cat: # 405214) antibody staining. In addition to the lineage markers for the analysis of LSK (Lin^−^Sca1^+^c-kit^+^) and LK (Lin^−^c-kit^+^) populations, fetal liver cells were stained with antibodies against Pacific Blue Pacific Blue-Sca1 (BioLegend, San Diego, CA; Cat #: 108120; Clone: D7), PeCy7-CD150 (BioLegend, San Diego, CA; Cat # 115914; Clone: TC15-12F12.2), APC-CD48 (eBioscience, San Diego, CA; Cat #: 17-0481-82; Clone: HM48-1) and eFluor780-c-Kit (eBioscience, San Diego, CA; Cat #: 47-1171-82; Clone: 2B8). Fetal liver cells were stained with PeCy7-CD16/32 (eBioscience; Cat #: 25-0161-82; Clone: 93) and Alexa Fluor 647 anti-CD34 (BD Biosciences, San Diego, CA; Cat #: 560230; Clone: RAM34) monoclonal antibodies to obtain GMP, CMP and MEP populations and with PE-IL7Ra (eBioscience, San Diego, CA; Cat #: 12-1271-82; Clone: A7R34) and CD135 PECy7 (eBioscience, San Diego, CA; Cat #: 12-1351-82; Clone: A2F10) to obtain CLP cells. Cell sorting was performed on MoFlo machines (Dako Cytomation) or FACS AriaII (Becton Dickinson, San Jose, CA).

For cell cycle analysis, surface marker-stained cells were fixed and permeabilized using Cytofix/Cytoperm buffer (BD Pharmingen, 554722) followed by intensive wash using Perm/Wash Buffer (BD Pharmingen, 554723). Cells were then labeled with Hoechst 33342 and Pyronin Y staining buffer (10 mg/mL Hoechst 33342 and 150 ng/mL Pyronin Y in Perm/Wash buffer; MilliporeSigma, 92-32-0) at 37˚C for 1 h at room temperature for 30 min followed by flow cytometry analysis on CD45.2^+^ SLAM-gated population.

For BrdU incorporation assay, Bromodeoxyuridine (BrdU, 150 μl of 10 mg/ml) were intraperitoneally (i.p.) injected to the indicated pregnant mice followed by embryo isolation 14 h later. BrdU incorporated cells (S phase) were analyzed with the BrdU Flow Kit (BD Biosciences, San Jose, CA; Cat #: 556029; Clone: 3D4), following the manufacturer’s instructions. Briefly, cells were surface stained then fixed and permeabilized using BD Cytofix/Cytoperm Buffer. After 1 h incubation with DNase at 37 °C, cells were stained with anti-BrdU monoclonal antibody. DAPI was added to each sample right before Flow Cytometry analysis (BD Biosciences, San Jose, CA).

For apoptosis staining, surface marker-stained cells were incubated with Annexin V and 7AAD using the BD ApoAlert Annexin V Kit (BD Pharmingen, San Jose, CA; Cat #: 550475) in accordance with the manufacturer’s instruction. Flow cytometry analysis was then performed to determine the proportion of Annexin V-positive cells.

For donor-derived chimera analysis, peripheral blood (PB) from the recipient mice were subjected to staining using PE-anti-CD45.1 and APC-anti-CD45.2 (both from BD Biosciences; Cat #: 553776 & 558702; Clone: A20 & 104) antibodies followed by flow cytometry analysis.

### Clonogenic assays

Single cell suspension from E12.5 fetal liver cells (1 × 10^5^) were plated in a 35-mm tissue culture dish in 3 mL of MethoCult M3134 (Stem Cell Technologies, Vancouver, BC, Canada) supplemented with the following growth factors: 100 ng/ml SCF, 10 ng/ml IL-3, 100 ng/ml GM-CSF, and 4 units/mL erythropoietin (Peprotech, Burlington, NC). On day 7 after plating, erythroid and myeloid colonies were enumerated. For serial plating, cells from primary CFU assays were pooled and re-plated to evaluate secondary CFUs [35]. Hematopoietic clonal growth results were expressed as means (of triplicate plates) ± SD of three experiments.

### Transplantations

8 ∼ 12-week-old BoyJ mice were used for all transplant experiments. For non-competitive BMT, 5 × 10^5^ fetal liver cells were injected via tail vein to lethally irradiated (11.75 Gy) BoyJ recipients. For competitive BMT, 5 × 10^5^ fetal liver cells, along with 5 × 10^5^ congenic BoyJ WBMCs were injected via tail vein. For secondary transplantations 3 ∼ 5 × 10^6^ BM cells from the primary recipients were injected to sublethally irradiated (7.0 Gy) BoyJ recipients.

### Immunohistochemistry and apoptosis analysis

Fetal livers were fixed overnight in 4% buffered formalin. Four-micron sections were produced from paraffin embedded samples and immunohistochemistry was performed according to antibody specification. Proliferation cells were detected with anti-ki67 antibody. Apoptotic cells were detected by cleaved caspase 3 antibody.

### Animal euthanasia and anesthesia

The method of euthanasia used in this study is CO2 narcosis before cervical dislocation. For all experiments described in this study, including tail biopsies, and tail bleeding, bleeding and pain is minimal, anesthesia is not required. This work has been reported in line with the ARRIVE guidelines 2.0.

## Results

### Loss of ***Hes1*** affects fetal development

Previous study has confirmed *Hes1* expression in mouse embryonic liver and spleen [36]. To study the role of HES1 in fetal hematopoiesis, we crossed the published *Hes1* conditional knockout mice (*Hes1*^*fl/fl*^; [4, 27]) with *Flt3Cre* strain, in which robust *Flt3Cre* activity begins in multipotent progenitors (MPPs) at E10.5 and is subsequently observed in more than 90% of mature leukocytes [29, 37]. We consistently observed a slightly reduced litter size from *Hes1*^*fl/fl*^*Flt3Cre* breeding compared to the control breeding (8.43 ± 1.5 vs. 6.43 ± 0.99; *p* = 0.0467), indicating that a small portion of embryos may have died during embryonic development. Due to the extremely low recombination activity of *Flt3Cre* in female mice [28, 37], *Hes1*^*fl/fl*^*Flt3Cre* females were born far below the expected Mendelian ratios (data not shown). Therefore, we decided to mate *Hes1*^*fl/fl*^*Flt3Cre* male with *Hes1*^*fl/fl*^ female for embryonic analysis (Fig S1A). Since Flt3 is generally considered to be absent from the surface of HSCs in the adult mice [38–40], *Hes1*^*fl/fl*^*Flt3Cre* male do not exhibit obvious phenotype (data not shown), which is consistent with our previous findings that HES1 is dispensable from adult hematopoiesis under steady state condition [4].

**Fig. 1:**
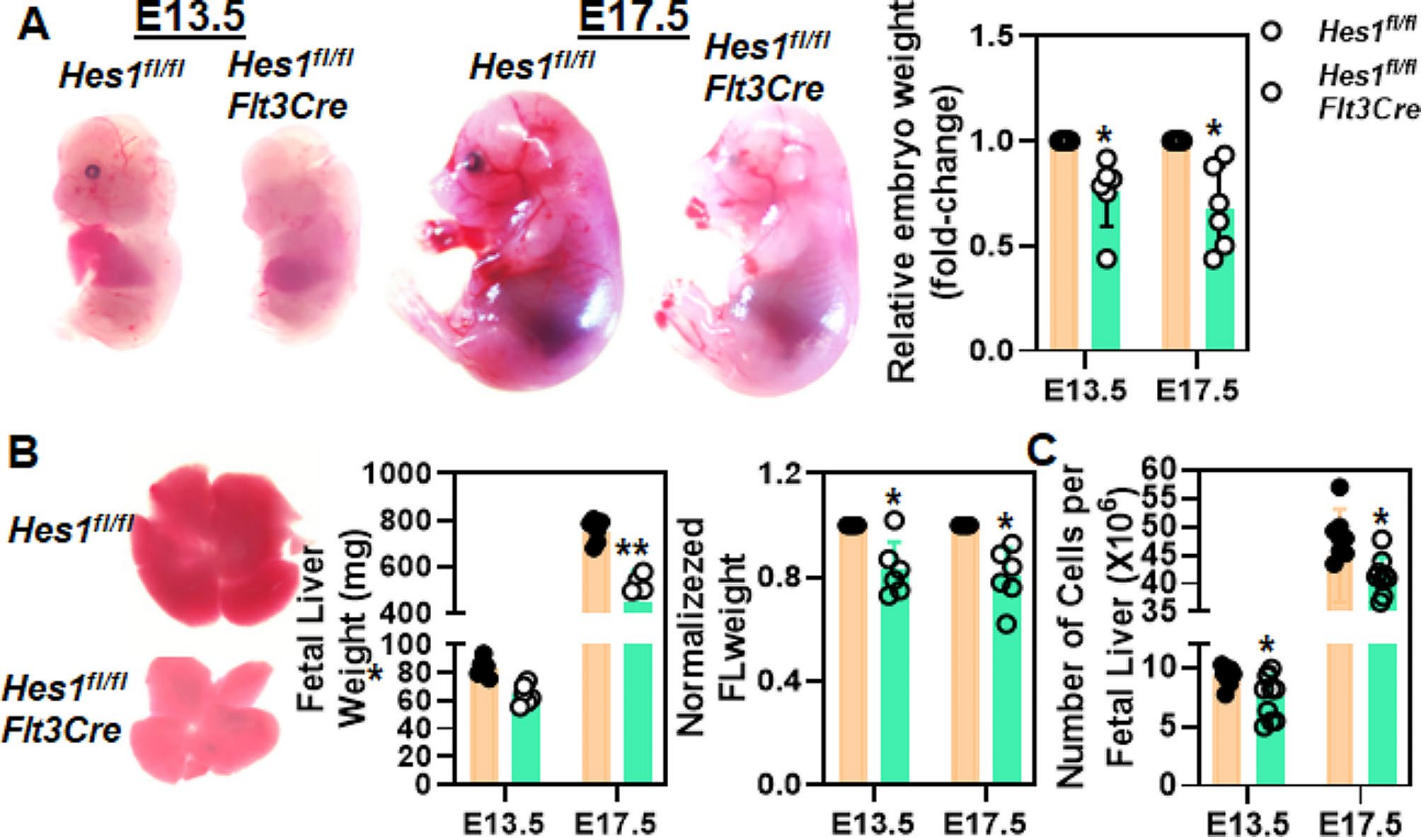
Loss of *Hes1* impairs embryonic development. (**A**) Smaller size of *Hes1*-KO embryos. Representative *Hes1*^*fl/fl*^ and *Hes1*^*fl/fl*^*Flt3Cre* embryo images at E13.5 and E17.5 (Left) and quantification of embryo weight (Right) are shown. (**B**) Loss of *Hes1* impairs fetal liver development. Representative dissected fetal livers from *Hes1*^*fl/fl*^ or *Hes1*^*fl/fl*^*Flt3Cre* embryos show reduced size and sometimes paler appearance of *Hes1*^*fl/fl*^*Flt3Cre* fetal livers. Quantifications of absolute (Middle) and normalized (to embryo body weight) fetal liver weight (Right) are shown. (**C**) Disruption of *Hes1* reduces total fetal liver cellularity. Fetal livers from E13.5 *Hes1*^*fl/fl*^*Flt3Cre* embryos contain dramatically reduced number of cells. Absolute fetal liver cell numbers (Left) and normalized data (Right) are shown. Results are means ± SD of three independent experiments (*n* = 6 ∼ 10 for each group)

Embryo genotypes determined by PCR analysis definitively identified *Hes1*^*fl/fl*^ (control) and *Hes1*^*fl/fl*^*Flt3Cre* (knockout; KO) embryos (Fig S1B). *Hes1* deletion in the fetal liver was confirmed by both immunohistochemistry (IHC) staining using fetal liver isolated on E13.5 (Fig S1C) and intracellular flow cytometry using fetal liver cells (Fig S1D). Gross examination of *Hes1*^*fl/fl*^*Flt3Cre* mutant embryos revealed that the most obvious consequence of homozygous knockout of *Hes1* gene was an impairment of embryonic development, evidenced by the reduction in the weight of *Hes1*^*fl/fl*^*Flt3Cre* embryos compared to the control embryos (Fig. 1A). It should be noted that hemizygous embryos appeared normal, consistent with the fact that healthy live births of hemizygous *Hes1*^*fl/+*^*Flt3Cre* males were obtained. Dissection of mutant embryos revealed that *Hes1* knockout results in smaller, anemic fetal livers, while the control fetal liver showed a bright red appearance (Fig. 1B, Left). *Hes1*^*fl/fl*^*Flt3Cre* fetal livers weighted significantly less than the control fetal livers (Fig. 1B, Middle), even after normalizing to total embryo weight (Fig. 1B, Right). In addition, the total cellularity of *Hes1*^*fl/fl*^*Flt3Cre* fetal liver was also slightly reduced (Fig. 1C). Together, these findings indicate that loss of *Hes1* affects fetal liver development.

### Loss of ***Hes1*** reduces HSPC pool and alters hematopoietic lineage differentiation

**Fig. 2:**
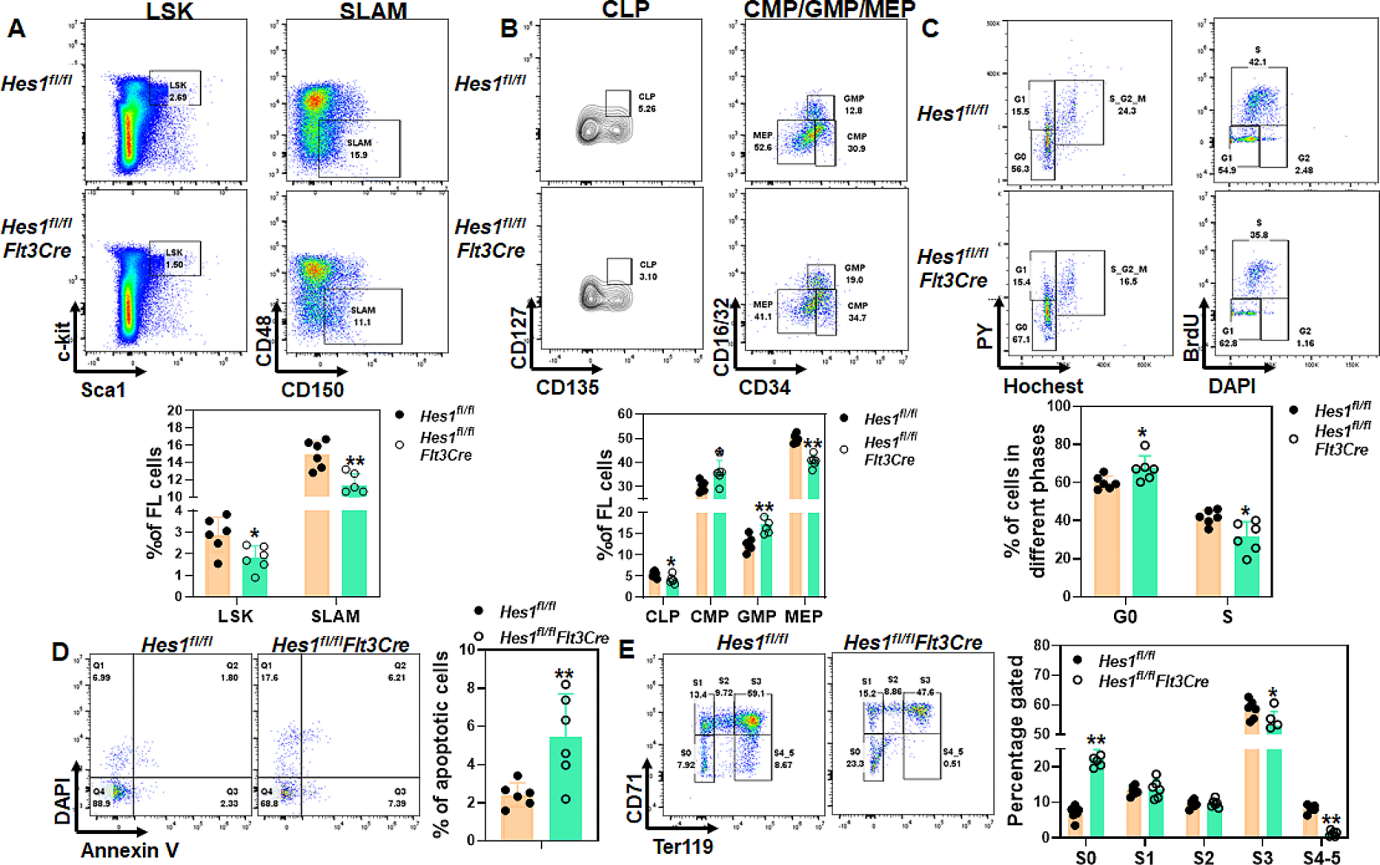
Loss of *Hes1* in fetal liver reduces embryonic HSPC pool. (**A**) Deletion of *Hes1* reduces HSPC pool in fetal liver. Representative flow plots for LSK, SLAM (Upper) and quantification (Lower) are shown. (**B**) Altered progenitor subpopulations in *Hes1*^*fl/fl*^*Flt3Cre* fetal livers. Representative flow plots for CLP, CMP, GMP and MEPs (Upper) and quantification (Lower) are shown. (**C**) *Hes1* deficiency limits HSPC cycling. Representative flow plots of Hochest/PY staining (Left) or BrdU incorporation (Right) and quantification (Lower) are shown. (**D**) *Hes1* deletion leads to increased apoptosis in fetal liver HSPCs. Representative flow plots (Left) and quantification (Right) are shown. (**E**) Loss of *Hes1* affects erythroid lineage differentiation. Representative flow plots (Left) and quantification (Right) are shown. Results are means ± SD of three independent experiments (*n* = 6 for each group)

We next performed flow cytometry analysis to examine different hematopoietic cell subpopulations in the mouse fetal livers. We found that *Hes1* deficiency caused a reduction in the frequencies of hematopoietic stem and progenitor cells (HSPCs, Lin^−^Sca1^+^c-kit^+^; LSK). Importantly, this reduction was even more profound in the phenotypic hematopoietic stem cell (HSC, Lin^−^Sca1^+^c-kit^+^CD150^+^CD48^−^; Signaling lymphocyte activation molecules; SLAM; 41) compartment (Fig. 2A). We also measured the frequences of other progenitor cells in the fetal liver [42, 43], namely CLP (common lymphoid progenitor), CMP (common myeloid progenitor), MEP (Megakaryocyte/erythrocyte progenitor), and GMP (granulocyte/macrophage progenitor). Flow cytometry analysis revealed that loss of *Hes1* resulted in increased CMP at the expense of CLP in *Hes1*^*fl/fl*^*Flt3Cre* fetal liver (Fig. 2B). Among the myeloid progenitors, we found that the frequencies of CMP and GMP were significantly higher, whereas MEP frequency was notably lower in *Hes1*^*fl/fl*^*Flt3Cre* fetal livers compared to those in the control fetal livers. These data suggest that deletion of *Hes1* in fetal hematopoietic system alters HSPC pool.

Quiescence is an important feature of HSC homeostasis [44–46]. Next, we analyzed the cell cycle status of fetal HSCs. Hochest 333342/Pyronin staining revealed a moderate increase in the proportion of quiescent (G0) SLAM cells in *Hes1*^*fl/fl*^*Flt3Cre* fetal liver compared to those in *Hes1*^*fl/fl*^ control livers (Fig. 2C, Left). Consistently, percentage of proliferating SLAM cells was notably lower in *Hes1*^*fl/fl*^*Flt3Cre* fetal liver than *Hes1*^*fl/fl*^ fetal livers as shown by a well-established BrdU incorporation assay (Fig. 2C, Right). In addition, we observed a slightly increased spontaneous apoptosis in SLAM cells isolated from *Hes1*^*fl/fl*^*Flt3Cre* fetal liver as analyzed by Annexin V/DAPI staining (Fig. 2D). These results suggest that HES1 may be required for fetal HSC homeostasis.

Since we observed anemic livers and increased MEP, indicative of a defective erythroid differentiation, in *Hes1*^*fl/fl*^*Flt3Cre* fetal livers, we then measured erythroid differentiation using the previously described CD71/Ter119 staining protocol [47–50]. We found that loss of *Hes1* led to an increase in S0 (CD71^intermediate^Ter119^low^) and decreases in S3 (CD71^high^Ter119^high^) and S4-5 (CD71^intermediate^Ter119^high^; Fig. 2E), indicating a blockage of S0 to S1 transition and an erythroid maturation defect. These data suggest that HES1 is required for lineage commitment during embryonic development.

### Loss of ***Hes1*** impairs HSPC function

**Fig. 3:**
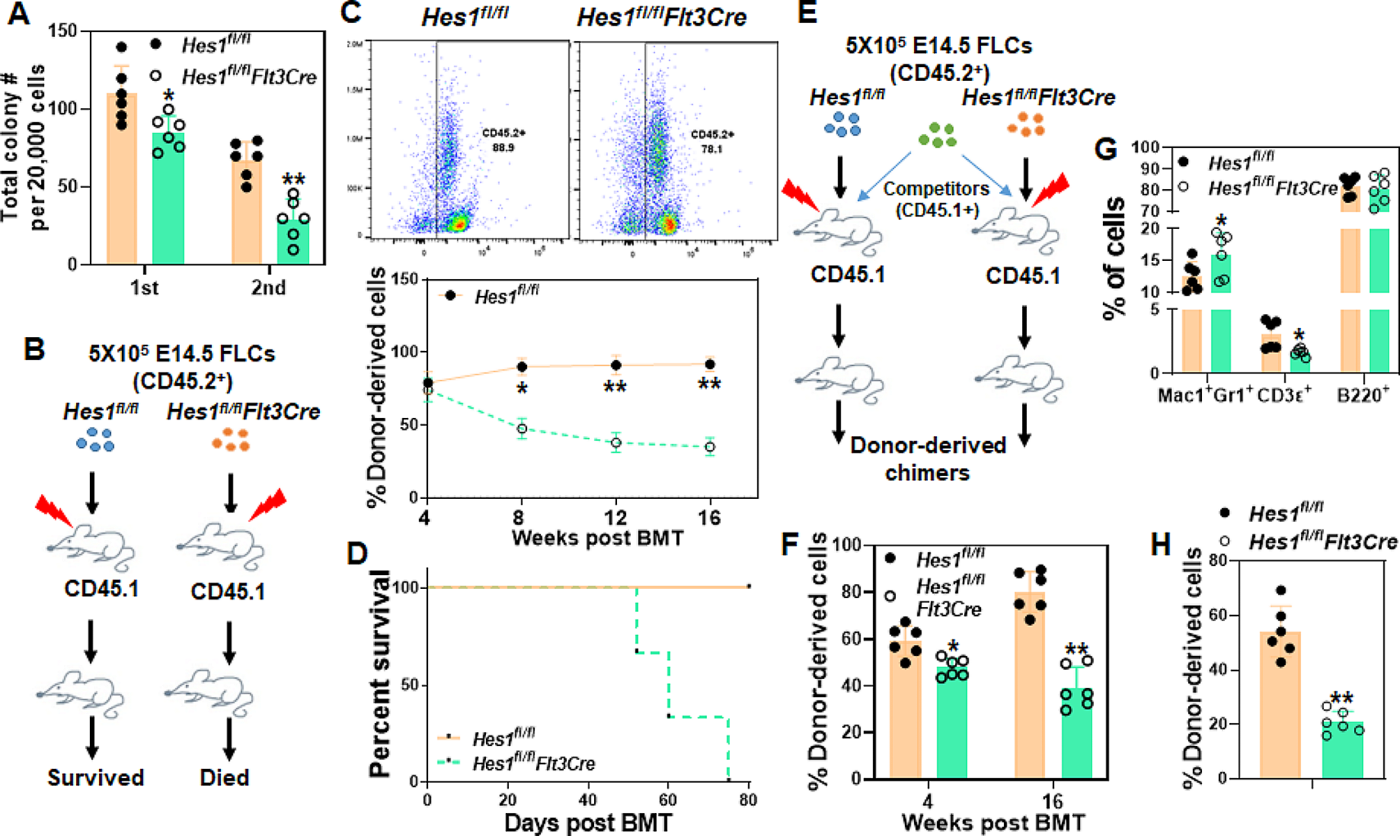
*Hes1* deletion compromises fetal HSPC function. (**A**) Deletion of *Hes1* in fetal hematopoietic cells leads to reduced progenitor activity. 2 × 10^5^ fetal liver cells from E12.5 *Hes1*^*fl/fl*^ or *Hes1*^*fl/fl*^*Flt3Cre* embryos were plated in cytokine supplemented methylcellulose medium. Colonies from the 1st plating were pooled for 2nd plating. Total colony number (Left) and different types of colonies (Right) were enumerated. (**B**) Schematic presentation of non-competitive BMT. 5 × 10^5^ fetal liver cells from E14.5 *Hes1*^*fl/fl*^ or *Hes1*^*fl/fl*^*Flt3Cre* embryos (CD45.2^+^) were transplanted into lethally irradiated BoyJ recipients (CD45.1^+^). 4 months later, whole bone marrow cells (WBMCs) from the primary recipients were transplanted into sublethally irradiated BoyJ recipients. (**C**) Loss of *Hes1* compromises hematopoietic reconstitution in the recipient mice. Donor-derived chimera in the primary recipients described in (**B**) was determined by flow cytometry at different time points post BMT. Representative flow plots at 4 weeks post BMT (Upper) and quantification (Lower) are shown. (**D**) *Hes1* deletion affects long-term repopulating capacity of fetal HSPCs. Survival of the 2nd recipients described in (**B**) are shown. (**E**) Schematic presentation of competitive BMT. 5 × 10^5^ fetal liver cells from *Hes1*^*fl/fl*^ or *Hes1*^*fl/fl*^*Flt3Cre* embryos (CD45.2^+^), along with 5 × 10^5^ congenic cells from BoyJ mice, were transplanted into lethally irradiated BoyJ recipients (CD45.1^+^). 4 months later, WBMCs from the primary recipients were transplanted into sublethally irradiated BoyJ recipients. (**F**) Loss of *Hes1* compromises hematopoietic reconstitution in the recipient mice. Donor-derived chimera described in (**E**) was measured by flow cytometry at 4 weeks and 16 weeks post BMT. (**G**) Loss of *Hes1* alters fetal HPSC differentiation. Donor-derived T, B and myeloid cells in the recipient mice described in (**E**) were measured by flow cytometry 16 weeks post BMT. (**H**) *Hes1* deletion affects long-term repopulation of fetal liver cells. Donor-derived chimera in 2nd recipients described in (**E**) was measured by flow cytometry 16 weeks post BMT. Results are means ± SD of three independent experiments (*n* = 6–9 for each group)

Decreased cycling and increased apoptosis of hematopoietic cells in *Hes1*^*fl/fl*^*Flt3Cre* fetal liver may affect HSPC function [45]. By using an in vitro colony formation unit (CFU) assay [46, 51], we found that fetal liver cells from E12.5 *Hes1*^*fl/fl*^*Flt3Cre* embryos produced significantly fewer colonies than those from *Hes1*^*fl/fl*^ embryos when plated in methylcellulose supplemented with hematopoietic cytokines (Fig. 3A, Left). Significantly, *Hes1*^*fl/fl*^*Flt3Cre* cells showed a marked decrease in serial replating activity compared to *Hes1*^*fl/fl*^ cells, indicative of replicative exhaustion in the absence of stromal support (Fig. 3A, Right).

We then measured the in vivo hematopoietic repopulating capacity of *Hes1*^*fl/fl*^*Flt3Cre* HSCs by a non-competitive bone marrow transplantation (BMT) assay (Fig. 3B), where 5 × 10^5^ E14.5 fetal liver cells from *Hes1*^*fl/fl*^ or *Hes1*^*fl/fl*^*Flt3Cre* embryos (CD45.2^+^) were injected into lethally irradiated BoyJ recipients (CD45.1^+^). Although we observed a significant reduction in donor-derived chimera in *Hes1*^*fl/fl*^*Flt3Cre* cell transplanted recipients, all the primary recipients survived 4-month observation period (Fig. 3C), albeit that the bones from the recipient mice transplanted with *Hes1*-KO fetal liver cells were paler than those transplanted with control cells (Fig S2A). Further analysis revealed that this reduction was not resulted from homing defects since *Hes1*-KO fetal liver cells gave rise to comparable CFSE^+^ cells in the bone marrow (BM) of the recipients 16 h post-transplant in a previously established homing assay (Fig S2B; [51, 52]). However, the secondary recipients transplanted with whole bone marrow cells (WBMCs) from *Hes1*^*fl/fl*^*Flt3Cre* fetal liver cells transplanted primary recipients died significantly earlier than those transplanted with WBMCs from the mice transplanted with control *Hes1*^*fl/fl*^ fetal liver cells (Fig. 3D), suggesting a premature HSC exhaustion.

To further substantiate the findings, we then performed competitive BMT (Fig. 3E) by transplanting 5 × 10^5^ E14.5 fetal liver cells from *Hes1*^*fl/fl*^ or *Hes1*^*fl/fl*^*Flt3Cre* fetal livers (CD45.2^+^), along with 5 × 10^5^ congenic WBMCs from the BoyJ mice into lethally irradiated BoyJ recipients (CD45.1^+^; 51). Flow cytometry analysis demonstrated a reduced donor-derived chimera (CD45.2^+^) in the peripheral blood (PB) of the recipients transplanted with *Hes1*^*fl/fl*^*Flt3Cre* fetal liver cells compared to those transplanted with *Hes1*^*fl/fl*^ cells (Fig. 3F). *Hes1* deletion also altered lineage differentiation (Fig. 3G), as evidenced by the slightly increased myeloid cells and decreased T cells in the recipients transplanted with *Hes1*^*fl/fl*^*Flt3Cre* fetal liver cells. Furthermore, complete blood count (CBC) revealed significant decreases in red blood cell (RBC) count, hemoglobin (Hb) and platelet count in the PB of *Hes1*^*fl/fl*^*Flt3Cre* fetal liver cells transplanted recipient mice compared with *Hes1*^*fl/fl*^ cells transplanted recipients (Fig S2C). Additionally, serial BMT confirmed a long-term repopulation defect of *Hes1*^*fl/fl*^*Flt3Cre* HSCs in the secondary transplanted recipients (Fig. 3H). Surprisingly, different from that *Hes1*-deficient adult HSCs undergo exhaustion under replicative stress through augmented fatty acid oxidative (FAO; 4), fetal LSK cells deficient for *Hes1* only exhibited marginally increased FAO (Fig S3A). Consistently, FAO related genes were scarcely upregulated (Fig S3B). Taken together, these data suggest that HES1 is required for fetal HSC maintenance, possibly through a distinct mechanism from their adult counterparts.

### Depletion of ***Hes1*** leads to decreased proliferation and increased apoptosis of fetal liver

**Fig. 4:**
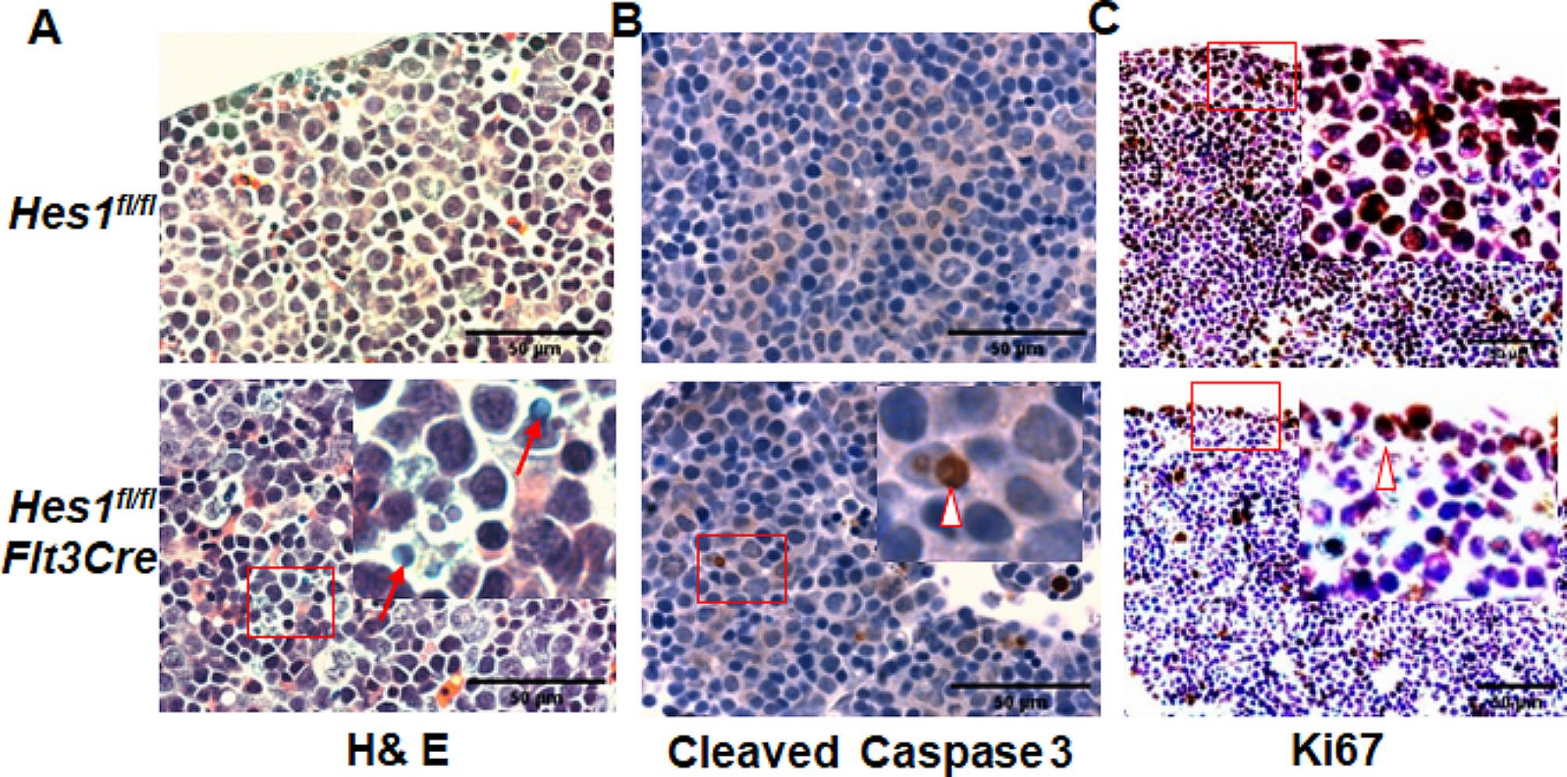
*Hes1*-deficient fetal liver exhibits reduced proliferation and increased apoptosis. (**A**) H&E-stained sagittal sections of fetal livers from *Hes1*^*fl/fl*^ or *Hes1*^*fl/fl*^*Flt3Cre* embryos reveal fragmented, pyknotic nuclei in *Hes1*^*fl/fl*^*Flt3Cre* embryo livers. (**B**) Loss of *Hes1* increases fetal liver cell apoptosis. *Hes1*^*fl/fl*^*Flt3Cre* fetal livers have increased cells stained positive for cleaved caspase 3 and fragmented nuclei compared with *Hes1*^*fl/fl*^ fetal livers, as seen in these representative E12.5 fetal liver sections stained with antibody to cleaved caspase 3 and counterstained with hematoxylin. (**C**) *Hes1*-KO fetal liver cells are less proliferative. *Hes1*^*fl/fl*^*Flt3Cre* fetal livers have fewer cells stained positive for Ki67 compared with *Hes1*^*fl/fl*^ fetal livers, as seen in these representative E12.5 fetal liver sections stained with antibody to Ki67 and counterstained with hematoxylin. The boxed area in the right corner of *Hes1*^*fl/fl*^*Flt3Cre* panel was magnified 50% and shown as an insert at top right of the same panel. Bar, 50 μm

Since *Hes1*-KO fetal livers appear to be smaller and anemic, we then performed histopathology analysis of fetal livers from *Hes1*^*fl/fl*^*Flt3Cre* or *Hes1*^*fl/fl*^ embryos. H&E-stained sagittal sections revealed fragmented pyknotic nuclei, which is the characteristic of cells undergoing apoptosis [53], in the fetal livers isolated from *Hes1*^*fl/fl*^*Flt3Cre* embryos (Fig. 4A). Moreover, *Hes1*^*fl/fl*^*Flt3Cre* fetal livers exhibited an increase in cells stained positive for cleaved caspase 3, a hallmark of both intrinsic and extrinsic apoptosis pathways ([54, 55]; Fig. 4B). These data support the notion that increased apoptosis contributes to the impaired development of *Hes1*^*fl/fl*^*Flt3Cre* fetal livers.

To determine whether the decrease in cell proliferation also contributes to the reduced size of *Hes1*^*fl/fl*^*Flt3Cre* fetal livers, we examined the level of cellular proliferation within the embryonic livers using the cell proliferation marker Ki67. Ki67 immunoreactivity of *Hes1*^*fl/fl*^ fetal livers showed that almost all cells were proliferating, consistent with rapid growth of the fetal liver at this stage of development (Fig. 4C, Upper; 9). However, Ki67^+^ cells in *Hes1*^*fl/fl*^*Flt3Cre* fetal livers were remarkably lower (Fig. 4C, Lower). Together, these results indicate that decreased fetal liver size in E13.5 *Hes1*^*fl/fl*^*Flt3Cre* embryos may be due to, at least in part, increased cell death and decreased proliferation.

### HES1 suppresses p27 and PTEN expression in the fetal liver

**Fig. 5:**
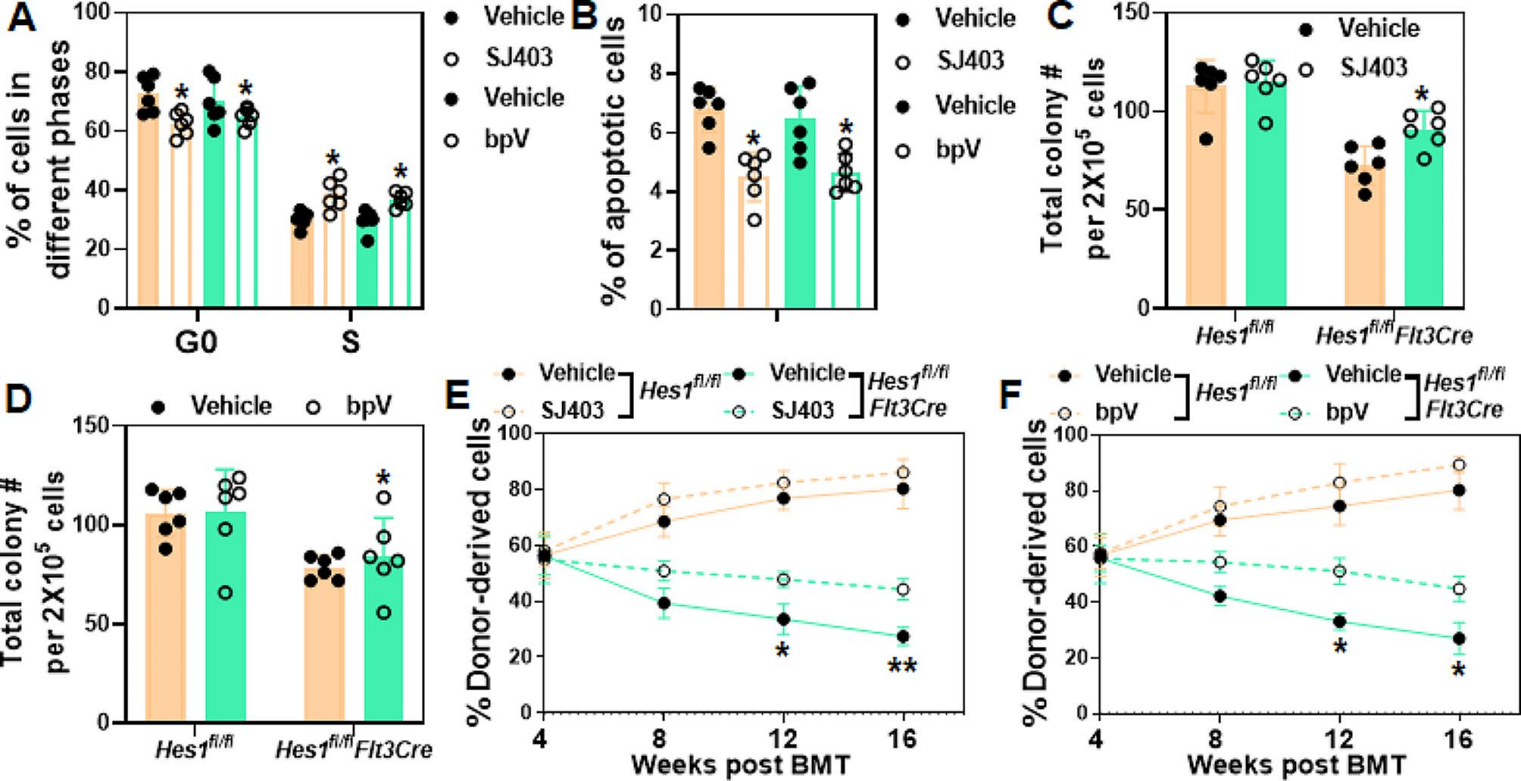
Pharmacological inhibition of p27 or PTEN rescues *Hes1*-deficient fetal HSPC function. (**A**) Pharmacological inhibition of p27 or PTEN improves HSPC cell proliferation. Fetal liver cells isolated from *Hes1*^*fl/fl*^*Flt3Cre* embryos were cultured in stem cell medium in the presence or absence of p27 or PTEN inhibitor (SJ403 or bpV, respectively) followed by flow cytometry analysis for Hochest/PY and BrdU incorporation, respectively. Quantifications of proportions of cells in G0 and S are shown. (**B**) p27 or PTEN inhibition reduces fetal HSPC cell death. Fetal HSPCs were cultured in stem cell medium in the presence or absence of p27 or PTEN inhibitor followed by flow cytometry analysis for apoptosis. (**C**, **D**) Inhibition of p27 or PTEN improves in vitro progenitor activity of *Hes1*-deficient fetal HSPCs. Fetal liver cells isolated from *Hes1*^*fl/fl*^*Flt3Cre* and *Hes1*^*fl/fl*^ embryos were cultured in stem cell medium in the presence or absence of p27 inhibitor (**C**) or PTEN inhibitor (**D**) followed by CFU assay. Colonies were enumerated on day 7. Quantifications are shown. (**E**, **F**) Pharmacological inhibition of p27 or PTEN rescues hematopoietic reconstitution defects of *Hes1*-KO fetal HSCs. Fetal liver cells isolated from *Hes1*^*fl/fl*^*Flt3Cre* and *Hes1*^*fl/fl*^ embryos were cultured in stem cell medium in the presence or absence of p27 (**E**) or PTEN inhibitor (**F**) followed by BMT transplantation to the lethally irradiated BoyJ recipients. Donor-derived chimera was measured at different time points post BMT. Results are means ± SD of three independent experiments (*n* = 6–9 for each group)

Previous studies have shown that HES1 directly controls cell proliferation through the transcriptional repression of p27^Kip1^ [24], a cyclin-dependent kinase (Cdk) inhibitor 1B that regulates cell proliferation, cell motility and apoptosis [56]. HES1 also opposes a PTEN-dependent check on survival, differentiation, and proliferation of TCRb-selected mouse thymocytes [25]. Since we observed reduced cell proliferation and increased cell death in *Hes1*-KO fetal liver, we then measured *Pten* and *p27* levels in HSPCs isolated from *Hes1*^*fl/fl*^*Flt3Cre* fetal liver. Indeed, we found that both mRNA and protein levels of PTEN and p27 were elevated in *Hes1*-deficient fetal HSPCs compared to those in the control HSPCs (Fig S4). Concomitantly, pharmacological inhibition of p27 by SJ572403 (SJ403; [31, 32]), or PTEN by bpV [33, 34], significantly improved HSC proliferation (Fig. 5A), and ameliorated apoptosis of mouse fetal HSPCs (Fig. 5B). Functionally, p27 and PTEN inhibition notably improved *Hes1*-KO progenitor activity in CFU assay (Fig. 5C and D), hematopoietic repopulating in the lethally irradiated recipient mice (Fig. 5E and F). Together, our data indicate that HES1 regulates fetal hematopoiesis through repressing p27 and PTEN, thereby promoting fetal cell proliferation and suppressing cell death.

## Discussion

Fetal hematopoiesis exhibits distinct features from adult hematopoiesis [9, 15, 57]. Although previous studies have shown the role of HES1 in regulating adult hematopoiesis, how HES1 functions in fetal hematopoiesis remains to be elucidated. By using a novel fetal hematopoietic specific *Hes1* knockout mouse model (*Hes1*^*fl/fl*^*Flt3Cre*), the study presented here provides evidence that HES1 is a critical regulator of fetal hematopoiesis in a cell autonomous manner. There are several findings that highlight the significance of our study: (1) HES1 is indispensable for fetal hematopoiesis; (2) *Hes1*-deficient mouse fetal HSCs are more quiescent and exhibit increased spontaneous apoptosis; (3) *Hes1* loss in fetal hematopoietic system affects lineage differentiation; (4) Deletion of *Hes1* de-represses *p27* and *Pten* and leads to elevated p27 and PTEN expression in fetal liver hematopoietic cells; (5) Pharmacological inhibition of p27 and PTEN improves *Hes1*-deficient fetal liver HSPC function.

It is known that fetal progenitors are highly proliferative, and have greater engraftment potential and higher propensity to form myeloid colonies compared with adult counterparts [16–21]. Here we show that HES1 plays distinct roles in fetal hematopoiesis and loss of *Hes1* in embryonic hematopoietic progenitor cells alters fetal liver HSPC subpopulation and compromises their function (Figs. 2, 3 and 4). While dispensable for steady state adult hematopoiesis, HES1 is required for stress hematopoiesis through regulating fatty acid oxidation (FAO; 4). One of the potential explanations on these disparities is that fetal HSPCs cope with highly proliferative status of fetal organ development, thereby are consistently under replication stress. Moreover, inflammatory signals play critical roles in fetal HSPC emergence, specification, and maturation [58]. In fact, each stage of pregnancy is characterized by a unique inflammatory environment [59]. A recent study demonstrated that HES1 acts in concert with a Fanconi anemia protein, FANCD2 to suppress inflammation-induced PPARg to prevent HSC exhaustion [60]. Our current data add another layer to the understanding of how HES1 regulates hematopoiesis during embryonic development, suggesting a distinct role for HES1 in modulating HSC maintenance at different stages of development.

In addition to previously described T cell development defects, here we show that Flt3Cre-mediated *Hes1* deletion impairs definitive hematopoiesis, particularly in erythroid lineage commitment (Fig. 2E and S2C). The haemopoietic composition of fetal liver during gestation shifts from being predominantly erythroid, accompanied by a parallel change in differentiation potential of HSC in human [61]. Similarly, three major waves of hematopoietic potential emerge sequentially during murine embryogenesis. First of all, primitive hematopoiesis emerges in the yolk sac and primitive erythroid cells subsequently mature in the bloodstream. Secondly, erythro-myeloid progenitors (EMP) emerge in the yolk sac then seed the fetal liver, where they rapidly differentiate and give rise to the first circulating definitive erythrocytes. These two waves of hematopoiesis also contain megakaryocyte and myeloid potential. Finally, adult repopulating HSCs emerge from major arteries and seed the fetal liver, and eventually the BM [62]. Mouse fetal liver between E11 and E15 is primarily an erythropoietic tissue [63]. CD71/Ter119 staining pattern in the fetal liver is dependent on embryonic age [48–50]. At the onset of definitive erythropoiesis on embryonic day 11 (E11) in the fetal liver, cells are concentrated in subsets S0 and S1, and are mostly erythroid colony-forming cells (CFU-e). With embryonic development, CFU-e cells differentiate into proerythroblasts (CD71^high^Ter119^intermediate^) and maturing erythroblasts and gradually populate subsets S2 to S5 [47–49]. Our current studies demonstrate that *Hes1* deficiency blocks fetal erythroid differentiation during S0 to S1 transition (Fig. 2E), which marks a switch from CFUe self-renewal to differentiation. Therefore, our findings lend support to the most recent study showing that another member of HES family member, HES6 regulates human erythropoiesis in a GATA1 and GATA2-independent manner [64, 65], and highlight a previously unappreciated role for HES family members in regulating erythropoiesis.

Another interesting finding of the present study is that loss of *Hes1* in fetal liver dampens HSC cycling and increases spontaneous cell death. We did not observe significant differences in HSC quiescence and apoptosis in adult hematopoiesis in *Hes1*^*fl/fl*^*Vav1Cre* mice [4]. The current findings suggest divergent roles for HES1 in regulating fetal versus adult hematopoiesis, which might be due to the higher expression of HES1 in fetal liver than in adult tissues [4, 36]. Mechanistically, we show that HES1 promotes proliferation and suppresses cell death through repressing p27 and PTEN expression in fetal HSPCs. Cyclin-dependent kinase inhibitor 1B, p27 is known as a mediator of the G1-S phase block [66]. Indeed, BrdU incorporation analysis demonstrates a decreased proportion of cells in S phase and increased proportion of cells stuck in G1 phase in *Hes1*-KO fetal liver (Fig. 2C), associated with the increased p27 in *Hes1*^*fl/fl*^*Flt3Cre* HSPCs (Fig S3). It has been shown that pharmacological inhibition of PTEN leads to PI3K/AKT/mTOR upregulation, and improves cell proliferation and migration [34, 67], thereby has been considered as a promising strategy for supporting tissue repair [68, 69]. In agreement with these findings, we show that PTEN inhibition significantly improves *Hes1*-deficient fetal HSPC function both in vitro and in vivo (Fig. 5). It is in this context, our study demonstrates that HES1 controls fetal hematopoiesis by two potential and synergistic cell autonomous mechanisms, subsequently modulates fetal HSPC viability and proliferation.

Hepatic and hematopoietic lineages develop simultaneously during the process of fetal liver hematopoiesis [9]. For example, macrophage-released oncostatin M (OSM) is crucial for hepatocyte differentiation during fetal hematopoietic development [70]. Similarly, megakaryocyte progenitors are known to promote hepatoepithelial cell development into hepatocytes in E11.5 fetal liver through a cell-to-cell contact manner [71]. Opposingly, hepatic stromal cells also affect fetal liver hematopoiesis [72]. In line with these previous reports, we observed fetal liver developmental defects in the newly established fetal hematopoietic specific *Hes1*-KO mice (Figs. 1 and 4). Although further analysis is needed, our present study highlights a bidirectional crosstalk between hepatocytes and hematopoietic cells during the embryonic development, emphasizing the role of embryonic hematopoietic cells in modulating fetal liver development.

## Conclusions

Our study identifies a previously undescribed role for HES1 in regulating definitive fetal hematopoiesis, and provide mechanistic insight into the function of HES1 in fetal HSC maintenance.

### Electronic supplementary material

Below is the link to the electronic supplementary material.

Supplementary Material 1

## Data Availability

All data generated or analyzed during this study are included in this published article.
