# Supplementary material for: HES1 is required for mouse fetal hematopoiesis
Source: Stem Cell Res Ther. 2024 Jul 29;15:235. doi: 10.1186/s13287-024-03836-8 (PMC11287931; doi:10.1186/s13287-024-03836-8)
Supplement: Supplementary file 1 — Supplementary Material 1 [file 13287_2024_3836_MOESM1_ESM.pdf]

Supplementary materials and methods

Homing assay

30,000 fetal liver LSK cells from E14.5 *Hes1*$^{fl/fl}$ or *Hes1*$^{fl/fl}$*Flt3Cre* embryos (CD45.2^{+}) were stained with 5 μM CFSE-mixed isomers (Thermo Fisher Scientific, Waltham, MA, USA) for 10 min according to the manufacturer's instructions (1, 2). The stained cells were re-suspended in 100 μl IMDM medium containing 30,000 cells and transplanted into lethally irradiated CD45.1 recipient mice (CD45.1^{+}). The mice were euthanized at 16 and 48 h after transplantation. WBMCs were analyzed by flow cytometry analysis for the presence of CFSE^{+} cells in PI^{-} cells in the BM from the recipient mice.

RNA extraction and qPCR analysis

Total RNA was extracted from FACS-sorted LSK cells (Lin^{-}Sca1^{+}c-kit^{+}) from E12.5 fetal liver using the RNeasy Micro Kit (QIAGEN, Germantown, MD), according to the manufacturer's instructions. RNA from whole fetal liver was isolated using the RNeasy Mini columns (QIAGEN, Germantown, MD). All RNAs were quantified with a Nanodrop 2000 spectrophotometer (Thermo Scientific, Waltham, MA) and assessed with an Agilent 2100 Bioanalyzer. First-strand cDNA was used for qPCR analysis using primers listed in Table S1. Samples were normalized to the level of *GAPDH* mRNA.

Measurement of fatty acid oxidation (FAO)

FAO was determined by palmitate oxidation method (3, 4). Briefly, metabolism of 1-^{14}C-palmitic acid (60 mCi/mmol; PerkinElmer, Waltham, MA) was determined as the formation of ^{14}C-acid-soluble β-oxidation products in LSK cells isolated from indicated mice. Cells

were permeabilized (10 μg digitonin/million cells), incubations contained 2 mM 1-^{14}C palmitate (10 nCi/assay) and the incubation lasted 15 minutes.

Statistical analysis

Paired or unpaired student's *t*-test was used for two-group comparison, and one-way ANOVA for more than two-group comparison. Values of *p* less than 0.05 were considered statistically significant. Results are presented as mean ± SD. * indicates p<0.05; ** indicates p<0.01; *** indicates p<0.001; **** indicates p<0.0001.

Graphpad Prism 9.0 was used for all statistical analysis. All data were checked for normal distribution and similar variance between groups. Data were derived from multiple independent experiments from distinct mice or cell culture plates. Sample sizes for *in vitro* studies were chosen based on observed effect sized and standard errors from prior studies. For all animal studies, a power test was used to determine the sample size needed to observe a twofold difference in means between groups with 0.8 power using a two-tailed Student's *t*-test. All animal studies were performed using sex- and age-matched animals, with wild-type littermates as controls. Animal studies were performed without blinding of the investigator, and no animals were excluded from the analysis. Statistics were performed in the indicated groups: 2-tailed, paired *t*-test (parametric) for two-group comparison and one-way ANOVA for more than two-group comparison. Normality of the data was tested with Shapiro-Wilk test. If the data is normally distributed, we used ANOVA followed by multiple comparisons with *t*-test. Otherwise, we used Kruskal-Wallis test followed by Wilcoxon rank sum tests. Values are reported as mean ± SD, unless stated otherwise.

References

1. Celso CL. Fleing HE, Wu JW, et al. Live-animal tracking of individual haematopoietic stem/progenitor cells in their niche. Nature. 2009;457:92-96.
2. Oostendorp RA, Audet J, Eaves CJ. High-resolution tracking of cell division suggests similar cell cycle kinetics of hematopoietic stem cells stimulated in vitro and in vivo. Blood. 2000;95:855-862.
3. Ma Z, Xu J, Wu L, Wang J, Lin Q, Chowdhury FA, Mazumder MHH, Hu G, Li X, Du W. Hes1 deficiency causes hematopoietic stem cell exhaustion. Stem Cells. 2020;38(6):756-768.
4. Huynh FK, Green MF, Koves TR, Hirschey MD. Measurement of fatty acid oxidation rates in animal tissues and cell lines. Methods Enzymol. 2014;542:391-405.

Supplementary table S1

Name	Forward	Reverse
Pten	TCCCAGACATGACAGCCATC	TGCTTTGAATCCAAAAACCTTACT
P27	AGCAGTGTCCAGGGATGAGGAA	TTCTTGGGCGTCTGCTCCACAG
Slc25a20	TTTGCAGGGATCTTCAACTG	CCCTTTGTACAAGGAGGTGA
Hadha	GTTTGAGGACCTCGGTGTAAAGC	GAGAGCAGATGTGTTGCTGGCA
Cpt1a	CATGTCAAGCCAGAGGAAGA	TGGTAGGAGAGCAGCACCTT
Cpt2	CAGCATATGATGGCTGAGTG	GTGGTTTATCCGCTGGTATG
Gapdh	TCAATGAAGGGGTCGTTGAT	CGTCCCGTAGACAAAATGGT

Supplementary figures

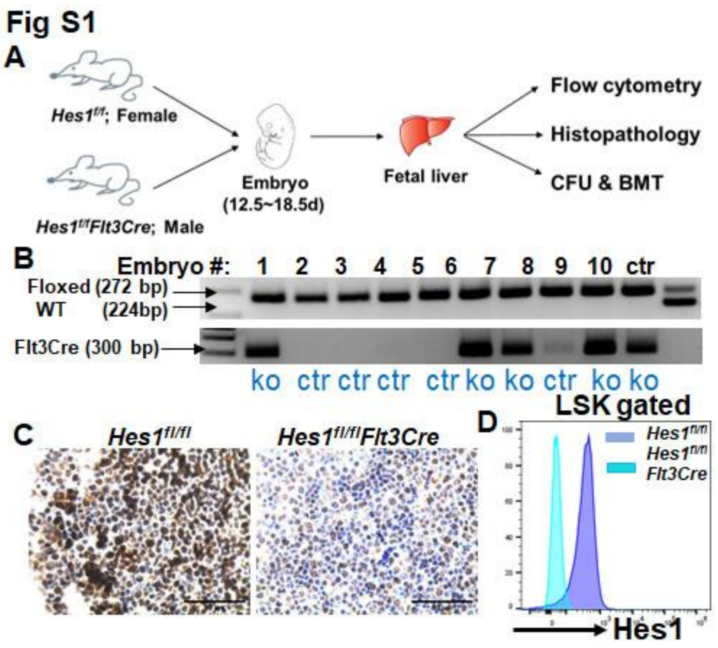

Fig S1. Deletion of *Hes1* in mouse fetal livers. (A) Schematic presentation of experimental design. (B) Genotyping identifies *Hes1$^{fl/fl}$* and *Hes1$^{fl/fl}$Flt3Cre* embryos. (C) Deletion efficiency of *Hes1* in E13.5 fetal liver. Immunohistochemistry (IHC) staining validate *Hes1* deletion in fetal liver. Representative E13.5 fetal liver sections stained with antibody to Hes1 and counterstained with hematoxylin. Bar, 50 μm. (D) Validation of *Hes1* deletion in fetal hematopoietic cells by flow cytometry. Fetal liver cells from *Hes1$^{fl/fl}$* and *Hes1$^{fl/fl}$Flt3Cre* embryos were subjected to intracellular HES1 staining followed by flow cytometry analysis. LSK (Lin^{-}Sca1^{+}c-kit^{+}) cells were gated for analysis.

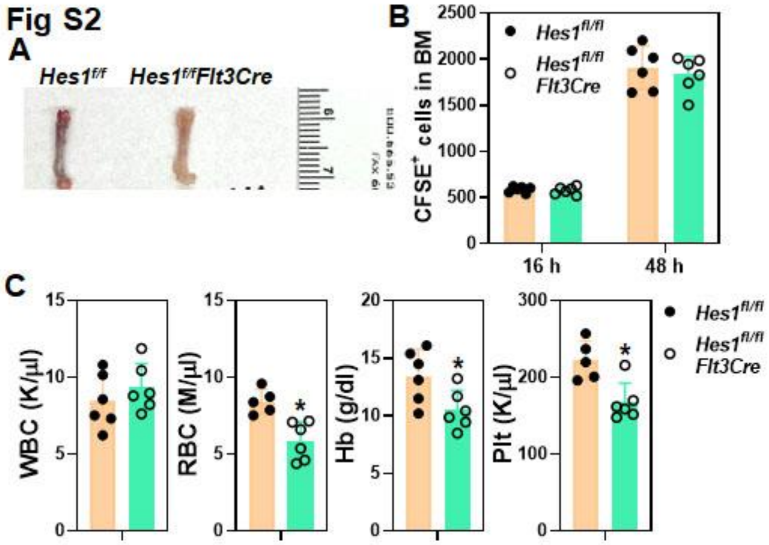

Fig S2. Loss of *Hes1* does not alter HSPC homing. (A) Paler femurs of the recipient mice transplanted with fetal liver cells into lethally irradiated BoyJ recipients. (B) Effect of *Hes1* deficiency on fetal HSPC homing. 30,000 CFSE labelled fetal liver cells from *Hes1*$^{fl/fl}$ or *Hes1*$^{fl/fl}$*Flt3Cre* embryos were transplanted into lethally irradiated CD45.1 recipient mice (CD45.1^{+}). WBMCs were analyzed by flow cytometry analysis for the presence of CFSE^{+} cells in PI^{-} cells in the BM from the recipient mice at 16 and 48 h after transplantation. (C) Peripheral blood parameters of the recipient mice described in Fig 3E.

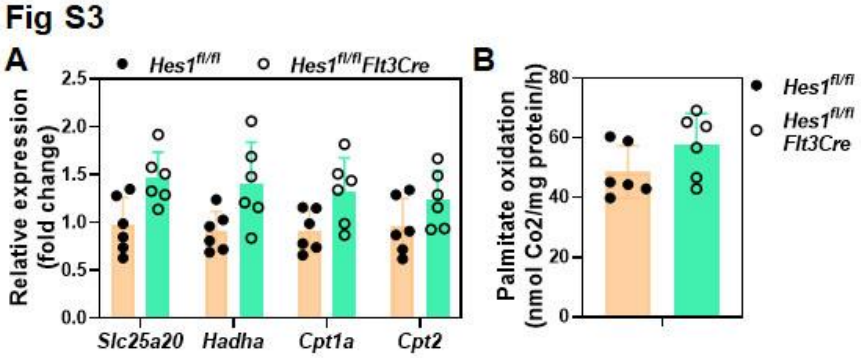

Fig S3. Deletion of *Hes1* in fetal liver does not affect FAO. A. Marginally increased FAO in *Hes1*-KO fetal liver hematopoietic cells. LSK (Lin$^-$Sca1$^+$c-kit$^+$) cells isolated from *Hes1*$^{fl/fl}$*Flt3Cre* or *Hes1*$^{fl/fl}$ fetal liver were subjected to palmitate oxidation rates measurement as captured $^{14}CO2$ using the isolated mitochondria and 1-^{14}C-palmitate as substrate. B. Fatty acid metabolism-related gene expression in *Hes1*$^{fl/fl}$*Flt3Cre* fetal LSK cells. RNA was extracted from LSK cells of *Hes1*$^{fl/fl}$*Flt3Cre* or *Hes1*$^{fl/fl}$ fetal liver followed by qPCR analysis using primers listed in Table S1. Samples were normalized to the level of GAPDH mRNA (n = 6). Quantifications are shown. Results are mean ± SD of three independent experiments. *p < .05; **p < .01

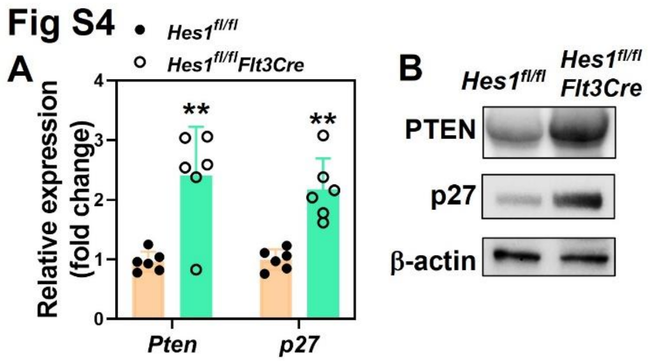

Fig S4. *Hes1* loss de-represses PTEN and P27 in fetal HSPCs. (A) Deletion of *Hes1* increases *Pten* and *p27* mRNA levels in fetal HSCs. mRNA extracted from SLAM cells from *Hes1*$^{fl/fl}$ or *Hes1*$^{fl/fl}$*Flt3Cre* fetal liver were subjected to qPCR analysis for *Pten* and *p27* using primers listed in Table S1. (B) *Hes1* loss leads to increased PTEN and P27 protein expression in fetal HSPCs. Proteins were extracted from Lin$^-$ cells isolated from *Hes1*$^{fl/fl}$ or *Hes1*$^{fl/fl}$*Flt3Cre* fetal liver, followed by immunoblots using antibodies against PTEN, p27 and β-actin.
